# Bed nets used to protect against malaria do not last long in a semi-arid area of Ethiopia: a cohort study

**DOI:** 10.1186/s12936-018-2391-5

**Published:** 2018-06-20

**Authors:** Tarekegn Solomon, Eskindir Loha, Wakgari Deressa, Meshesha Balkew, Taye Gari, Hans J. Overgaard, Bernt Lindtjørn

**Affiliations:** 1 School of Public and Environmental Health, Hawassa University, Hawassa, Ethiopia; 2 Department of Preventive Medicine, School of Public Health, College of Health Sciences, Addis Ababa University, Addis Ababa, Ethiopia; 3 Aklilu Lemma Institute of Pathobiology, Addis Ababa University, Addis Ababa, Ethiopia; 4 Norwegian University of Life Sciences, Ås, Norway; 5 Centre for International Health, University of Bergen, Bergen, Norway

**Keywords:** Durability, Long-lasting insecticide nets, Attrition, Physical integrity, Functional survivorship, Ethiopia

## Abstract

**Background:**

Long-lasting insecticidal nets (LLINs) are a key tool for malaria prevention and control. Currently, the recommended serviceable life of an LLIN is 3 years under field conditions. However, field studies show considerable variation in LLIN lifespan, from less than 2 years to more than 4 years. This study aimed to determine the attrition, physical integrity, functional survival, and bio-efficacy of LLINs under field conditions in south-central Ethiopia.

**Methods:**

In October 2014, 7740 LLINs (PermaNet^®^ 2.0) were distributed to 3006 households. Among the distributed LLINs, a cohort study involving 1532 LLINs in 659 households was carried out from October 2014 to November 2016. Data were collected every 6 months by observation, and through interviews with the heads of households. The proportional hole index was used to categorize LLINs as either serviceable or torn. In addition, 120 randomly selected LLINs were tested for bio-efficacy.

**Results:**

The overall attrition of LLINs was 96% (n = 993) during the study period. The nets’ attrition was mainly due to disposal (64.2%; n = 638). The proportion of LLINs with a hole size 0.5 cm or larger was 79.5% after 24 months. The use of the net on the previous night and having a clean net were associated with a good physical integrity. However, living in a household more than 1 km away from the mosquitoes’ breeding site was associated with poor physical integrity. By the 24th month, only 4% of the nets met the criteria for functional survival. The median functional survival time of the nets was 12 months. A longer functional survival was associated with having a clean net, and shorter survival was associated with living in a household more than 1 km away from the mosquitoes’ breeding site. The PermaNet^®^ 2.0 met the criteria of effective bio-efficacy up to month 24 after distribution.

**Conclusions:**

The study showed that the median serviceable life of LLINs is only 12 months. However, the bio-efficacy of the LLINs is acceptable for at least 24 months. Therefore, stronger and more efficient LLINs need to be developed for conditions similar to those studied here.

## Background

Globally, the burden of malaria has declined in the past 15 years with the scaling-up of cost-effective vector control interventions, diagnosis, and treatment [1]. The reduction in the global incidence of malaria is estimated to be 37%, and the decline in malaria-specific mortality is estimated to be 60% [1]. Similar reductions have also been observed in Ethiopia [2], but the incidence of malaria is still high; it is estimated that 2,588,000 cases of malaria and 5000 malaria-specific deaths occurred in 2016 [3].

Vector control through the use of long-lasting insecticidal nets (LLINs) is a widely implemented tool for the prevention of malaria [4, 5]. To maximize the impact of the intervention, universal access to and use of LLINs by people at risk for malaria must be maintained [6]. However, access to LLINs remains lower than expected [3]. For example, in sub-Saharan Africa, only 43% of people had access to sufficient LLINs (a net for two people), and only 54% people slept under LLINs in 2016 [3]. According to a 2015 national malaria indicator survey, 64% of Ethiopian households own at least one LLIN, and 32% have one LLIN for every two persons. The same survey reported that only 40% of the population at risk slept under a LLIN the night before the survey [7].

The LLIN interventions have a limited service life because they become worn out or are lost. The most common causes for the short service life of LLINs are a high attrition rate and physical damage [8–12]. Moreover, care and repair of bed nets, usage pattern, washing frequency, and type of LLIN all have potential impacts on the length of the service life of an LLIN [8, 13–15]. The World Health Organization (WHO) recommends that LLINs should be serviceable for at least 3 years under field conditions, with adequate insecticidal activity [16]. However, studies show considerable variation in the length of an LLIN’ serviceable life, ranging from less than 2 years to more than 4 years [8, 13, 17–19]. Furthermore, it cannot be assumed that an LLIN product that is durable in one setting will last in other settings. Thus, there is substantial need for regional data to assess the durability of LLINs [20, 21]. Such data could inform decision-makers regarding how often bed nets should be distributed. Furthermore, understanding the factors that lead to a shortened LLIN service life could help guide communication interventions for behavioural change [20].

Previous studies from Ethiopia have investigated physical integrity and bio-efficacy of LLINs using cross-sectional study design [17, 21, 22]. However, these studies did not consider the attrition rate, functional survival, or potential causes of poor physical integrity of LLINs. To fill this knowledge gap, this study used a cohort design to determine the durability of LLINs under user conditions in the Adami Tullu district, south-central Ethiopia, in terms of attrition, physical integrity, functional survival, and bio-efficacy.

## Methods

### Study setting

**Fig. 1:**
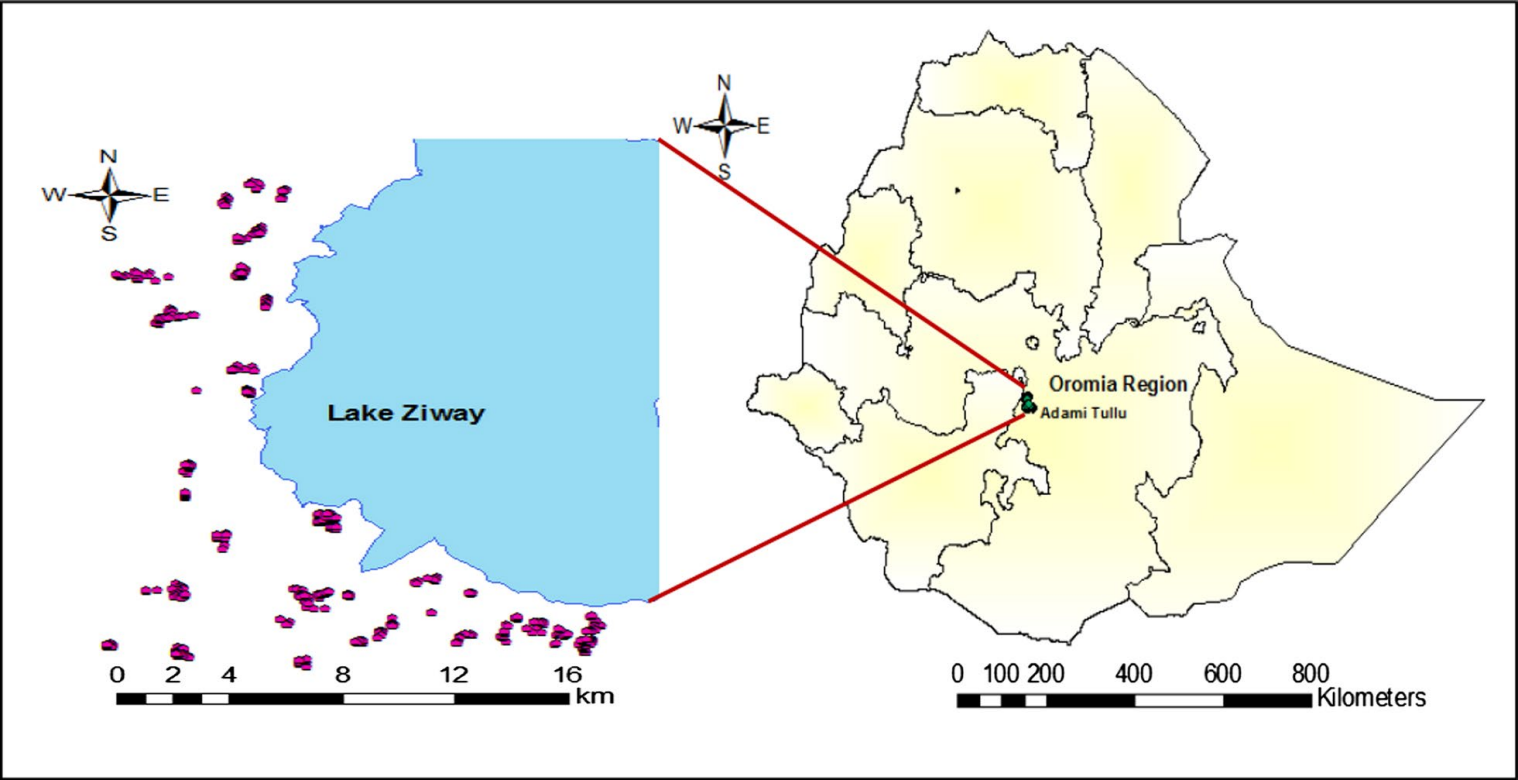
Map of the study area showing the location of selected households in south-central Ethiopia

This study was carried out in the Adami Tullu district in south-central Ethiopia (Fig. 1) from October 2014 to November 2016. The district is located approximately 160 km south of Addis Ababa. The study population was primarily composed of the Oromo ethnic group, who follow the religion Islam. This rural population primarily engages in farming, livestock, and fishing. Based on the 2007 national census, approximately 190,000 people lived in the district in 2017 [23]. The district has 48 *kebeles* (the lowest government administrative unit), each with an average population ranging from 1000 to 5000 people [23].

Malaria is among the leading causes of illness in the Adami Tullu district, sometimes occurring as an epidemic [24]. The shores of Lake Zeway and irrigated areas serve as mosquito breeding sites in the district [25, 26]. *Anopheles arabiensis* is the main malaria vector, whereas *Plasmodium falciparum* and *Plasmodium vivax* are the main parasites of malaria in the district [27, 28]. The district is a drought-prone area, and is characterized by a semi-arid climatic condition [29]. The district was affected by a severe drought that occurred in 2015 following the El Nino [30]. Effects of the drought included food shortages, a decline in annual rainfall (by 60% in 2015) and an increase in the average maximum temperature (2 °C above normal) [31].

### Study design and participants

This study was part of a cluster-randomized controlled trial that aimed at quantifying the combined effect of indoor residual spray (IRS) and LLINs against clinical malaria, compared with LLINs or IRS alone or routine intervention (the MalTrials project) [32]. The trial had 176 study clusters (44 clusters per arm). The LLIN + IRS arm contained 1619 households and 8216 people; the LLIN-alone arm contained 1387 households and 7288 people; the IRS-alone arm contained 1530 households and 7753 people; and the routine arm contained 1544 households and 8038 people. In early October 2014, 7740 LLINs (purchased in June 2014 from the Vestergaard Frandsen Group) were distributed to 3006 households, both in combination and in the LLIN-alone arm. They had a light blue colour and rectangular shape, with a size of 160 cm width, 180 cm length, and 150 cm height [32]. The distribution of LLINs was conducted based on the National Malaria Guidelines: one net for a family with 1–2 persons; two nets for a family with 3–5 persons; three nets for a family with 6–7 persons; and four nets for a family with ≥ 8 persons [33]. A “hang-up” campaign and net tracking activities were carried out after distribution by putting a unique identification number on each LLIN with indelible ink.

Using the malaria trial framework [32], a cohort study was conducted among households with newly distributed LLINs to assess attrition, physical integrity, and functional survivorship. Four follow-up surveys were conducted every 6 months. The first survey was conducted in April 2015, the second in October 2015, the third in April 2016, and the fourth in early November 2016. The LLINs were followed until one of the following outcomes: LLIN loss due to discarding, distraction, used for other purposes, given away to other users, sold, stolen, lost to follow up, torn, or to the end of the study. Moreover, every 6 months cross-sectional surveys were carried out to evaluate the ability of LLINs to knockdown (KD) or kill susceptible *Anopheles* mosquitoes (bio-efficacy).

### Sample size estimation

The sample size was calculated based on the findings from a study in Benin, in which 48% of the LLINs were in poor condition (torn) after 1.5 years of use [13]. Using a single-population proportion formula (with OpenEpi software), and assuming a 4% margin of error, a 95% confidence level at α = 5%, and a 10% non-response rate, a total sample size of 659 LLINs was calculated. The households were randomly selected from a sampling frame of the LLIN-alone arm of the trial. Computer-generated random numbers were used to select random samples of LLINs using IBM SPSS version 20.0 (Armonk, NY: IBM Corp. USA). To avoid selection bias, all LLINs in the selected households were included in the study. A total of 1532 LLINs in 659 households were enrolled at baseline for evaluation of attrition and functional survivorship. A sub-sample of 833 LLINs were enrolled at the sixth month and followed for assessment of physical integrity. According to WHO recommendations [34], a total of 120 LLINs (30 LLINs per survey) were evaluated for bio-efficacy over a 2-year period. The LLINs were collected for the test based on the eligibility criteria of being used for sleeping during data collection. One LLIN per household was considered for the test. The LLINs that were taken for analysis were immediately replaced with new LLINs.

### Data collection

Baseline and follow-up data on household characteristics and net status were collected using structured, pre-tested and interviewer-administered questionnaires. The questionnaires were prepared in English and then translated into the local language, *Afan Oromo*. The USAID-supported Malaria Consortium NetWorks training guideline was used to train data collectors for 2 days on the LLIN hole assessment technique [35]. All data collectors were diploma graduate personnel. Three teams of data collectors, each of which was comprised of three members (one supervisor and two data collectors in each team), were involved in data collection. During data collection, heads of households or competent family members (age ≥ 18 years) were interviewed about the status of their LLINs. If LLINs were not found or used for other purposes, the respondents were asked why and how nets were lost or damaged or used for other purposes. If the visited house was closed or no competent (age ≥ 18 years) respondent was present, the house was revisited at least three times within a week. If the house was closed or no competent respondent was present after three visits, LLINs were considered lost to follow-up.

### Definition and follow up of outcome variables

#### Attrition

Attrition was defined as the proportion of LLINs no longer in household use [34]. Attrition of LLINs was categorized as “attrition for known outcome” and “attrition for unknown outcome”.

#### Attrition for known outcome

Net lost from household due to discarding, destruction, or used for other purposes.

#### Attrition for unknown outcome

Net lost from household due to being given away for others to use, used in different location, stolen, sold or lost to follow up (due to family moving to other location or not at home).

#### Physical integrity

The physical integrity of the LLINs was defined considering the number, size, and location of holes to estimate the protection ability of the net against mosquito bites. For nets presented and used for sleeping during data collection, inspections were carried out for the presence, type, location, and size of holes. A rectangular metal frame with a size of 165 cm width, 185 cm length, and 160 cm height was used to hang and inspect each net for holes. Hole categories recommended by the WHO were used to determine hole size [20]. Hole-size categories were defined as follows: hole size 1, 0.5–2 cm (smaller than a thumb); hole size 2, 2–10 cm (larger than a thumb, but smaller than a fist); hole size 3, 10–25 cm (larger than a fist, but smaller than a head); and hole size 4, larger than 25 cm (larger than a head). Holes smaller than 0.5 cm were not counted. Moreover, the causes of holes were identified and evidence of repair was recorded. The proportional hole index (pHI) was used to group LLINs into serviceable or torn categories. The pHI for each LLIN was calculated by weighting each hole by its size (size 1–4) and totaling up the weighted number of holes as described elsewhere [34]. The LLINs with holes were categorized into one of the following groups: pHI 0–64, “good condition”: no reduction of efficacy compared to an undamaged net; pHI 65–642, “acceptable condition”: effectiveness somewhat reduced, but still provides significantly more protection than no net at all; and pHI ≥ 643, “torn” or poor physical integrity condition: the protective efficacy is in serious doubt, and the LLIN should be replaced as quickly as possible. The number of combined LLINs in “good” and “acceptable” condition represented the number of LLINs in “serviceable” condition or in good physical integrity condition [34].

#### Functional survivorship

Functional survival was defined as the proportion of LLINs in serviceable (“good” + “acceptable”) condition at a given time point after LLIN distribution. Both attrition with known outcome and LLINs in serviceable or torn conditions were used to evaluate functional survival [36].

#### Bio-efficacy

The ability of a net to incapacitate or kill susceptible *Anopheles* mosquitoes after contact with the insecticide on the LLIN. For the bio-efficacy test, five samples from each LLIN measuring 30 cm × 30 cm were cut according to the guideline [34]. Each piece of the net section was labelled with a unique identification number by combining the household number and a sample location. The samples were then wrapped in a foil and placed in a black plastic bag for storage until the test. In the laboratory, 10 susceptible, 2- to 5-day-old, non-blood fed female *An. arabiensis* mosquitoes were exposed for 3 min on each piece of sample according to the WHO cone bioassay test procedure [12]. Control tests were carried out each day immediately before and after exposure of mosquitoes to experimental LLINs. The LLINs fulfilling the criteria of ≥ 95% KD or ≥ 80% mortality using susceptible *Anopheles* mosquitoes were considered effective [34].

### Statistical analysis

Data were entered into and analysed by IBM SPSS version 20.0. For non-normally distributed continuous variables, medians and the interquartile range (IQR) were calculated. The dependent variables of the study were attrition, physical integrity, functional survival, and the bio-efficacy of LLINs. The exposure variables were gender and educational status of the head of the household, family size, wealth status, the presence of open eave gaps in the house, type of bed, status of net use, status of net washing, hygienic condition of the LLIN, presence of rodents or cats in the household and distance of household from vector breeding sites.

The household wealth index was calculated using principal component analysis (PCA) [37, 38]. Fourteen household assets were used in the calculations, including presence of electricity, ownership of television, radio, mobile telephone, chair, table, bed, bicycle, land, separate kitchen from living house, animal and animal cart, and types of roof and walls. A wealth index was constructed from the first principal component for each household, and then categorized into three relative measures of socioeconomic class (poor, middle, and rich). The Kaiser-Meyer-Olkin (KMO) measure of sample adequacy was 0.77. The total variance explained by the first principal component was 23.8%, with a corresponding Eigen value of 3.33.

The attrition rate of the LLINs was calculated as the number of LLINs lost with known outcome of attrition or torn, divided by all LLINs enrolled at baseline. However, the LLINs lost with unknown outcome of attrition were excluded from the denominator. The physical integrity of the net was determined using two measurements. The first measurement was the proportion of LLINs with a hole size 0.5 cm or larger divided by the total number of coded LLINs found and assessed in the surveyed households. The second measurement was the proportion of torn nets divided by all nets assessed for holes. To estimate the proportion of functionally surviving LLINs, the nets in “good” and “acceptable” condition were used as a numerator, and all nets present in surveyed households and nets lost due to “known outcome of attrition” + torn were used as a denominator. The proportion of functionally surviving nets was compared against reference survival curves provided by the WHO [36]. A Kaplan–Meier survival analysis was used to estimate the median survival time of functionally surviving LLINs. The proportion of LLINs with effective bio-efficacy was calculated as the number of effective LLINs (≥ 95% KD or ≥ 80% mortality) divided by the total number of LLINs tested. The LLINs were considered effective against malaria-transmitting *Anopheles* mosquitoes if at least 80% of the sampled LLINs fulfilled the criteria of ≥ 95% KD or ≥ 80% mortality after at least 20 washes and 3 years of use [34].

To investigate the predictors of physical integrity and functional survival of LLINs, a proportional Cox regression model was fitted to the dataset. The failure endpoint for physical integrity was defined as an LLIN in torn condition. And, for functional survival, the failure endpoint was either the LLIN having the known outcome of attrition or being in torn condition. The LLINs in the unknown outcome of attrition category were censored at the time of net loss. Variables having a P value < 0.25 in bivariate analysis were included in the multivariate analysis to identify independent predictors. A P value < 0.05 was considered statistically significant.

### Ethical considerations

Ethical clearance was obtained from the Ethiopian Ministry of Science and Technology (Ref: 3.10/446/06), Institutional Review Board of the College of Health Sciences at Addis Ababa University and the Regional Committee for Medical and Health Research Ethics, Western Norway (Ref: 2013/986/REK vest). Also, permission letters were obtained from the Oromia Regional Health Bureau, East Shewa Zonal Health Department, and Adami Tullu District Health Office. Information about the study objectives, procedures and benefits were clearly explained to the study participants. Written consent was not obtained because the majority of the study participants could not read or write [32]. Therefore, verbal informed consent was obtained from study participants during data collection.

## Results

### Characteristics of study households

A total of 659 households were included in this study. The majority of heads of households were male (407; 62%) and illiterate (369; 57%). About 331 (50%) of study households had a family size of more than five individuals. Approximately one-third of households (202; 31%) lived within 1 km from a potential vector breeding site (Table 1).

**Table 1:** Characteristics of households with long-lasting insecticidal nets assessed for durability in Ethiopia

Variable	n (%)
Gender of head of household	
Male	407 (61.8)
Female	252 (38.2)
Educational status of head of household (n = 647)	
Illiterate	369 (57.0)
Read and write	59 (9.1)
Primary	162 (25.0)
Secondary and above	57 (8.8)
Wealth status (n = 622)	
Poor	230 (37.0)
Middle	198 (31.8)
Rich	194 (31.2)
Family size	
≤ 5	328 (49.8)
> 5	331 (50.2)
House with open eave gap (n = 615)	
Yes	99 (16.1)
No	516 (83.9)
Distance from mosquito breeding site (km)	
≤ 1	202 (30.7)
> 1	457 (69.3)

### Enrollment of LLINs and study completion

At the start of the study, 1532 LLINs were included in the study for attrition and functional survival assessment. Out of this number, 1061 at 6 months (T_6_), 517 at 12 months (T_12_), 198 at 18 months (T_18_), and 56 at 24 months (T_24_) were available for examination.

### Attrition

**Fig. 2:**
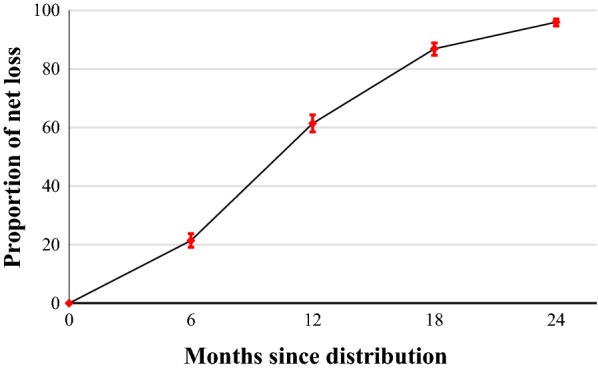
Proportion of lost LLINs due to known reasons of attrition or torn over a 2-year-period. The error bars indicate the 95% confidence interval

A total of 1491 LLINs were lost during the 2-year follow-up period. Among the lost LLINs, 993 (67%) were lost due to a known outcome or torn, and 498 (33%) were lost due to an unknown outcome (Table 2). The attrition for known outcomes or torn of LLINs increased more rapidly over time (Fig. 2). The overall attrition for known outcomes or torn from the beginning to the end of the study was 96% (95% CI 94.7–97.1; n = 993; N = 1034). The reasons for this attrition were as follows: being thrown away because of damage (638; 64.2%), torn (217; 21.9%), and being used for other purposes (138; 13.9%; Table 2).

**Table 2:** Reasons for loss of long-lasting insecticide nets over a 2-year follow-up period in Ethiopia

Reason for LLIN loss	6 months n (%)	12 months n (%)	18 months n (%)	24 months n (%)	Total n (%)
Known outcome of attrition or torn					
Thrown away	102 (40.0)	301 (71.8)	173 (74.9)	62 (70.5)	638 (64.2)
Used for something else	30 (11.8)	58 (13.8)	28 (12.1)	22 (25.0)	138 (13.9)
Torn (pHI > 643)	123 (48.2)	60 (14.3)	30 (13.0)	4 (4.5)	217 (21.9)
Total	161 (100)	331.1 (100)	149 (100)	12 (100)	993 (100)
Unknown outcome of attrition					
Given away	260 (76.7)	44 (45.8)	11 (20.0)	1 (12.5)	316 (63.5)
Lost to follow-up^a^	52 (15.3)	43 (44.8)	26 (47.2)	5 (62.5)	126 (25.3)
Stolen	8 (2.4)	9 (9.4)	3 (5.5)	2 (25.0)	22 (4.4)
Unknown reasons	6 (1.8)	0 (0.0)	1 (1.8)	0 (0.0)	7 (1.4)
Other^b^	13 (3.8)	0 (0.0)	14 (25.5)	0 (0.0)	27 (5.4)
Total	461 (100)	558.2 (100)	153 (100)	(168) (100)	498 (100)

*LLIN* long-lasting insecticidal net

^a^ Family moved to other location, family not at home, refusal to participate

^b^ Sold or destroyed by fire

### Physical integrity

**Fig. 3:**
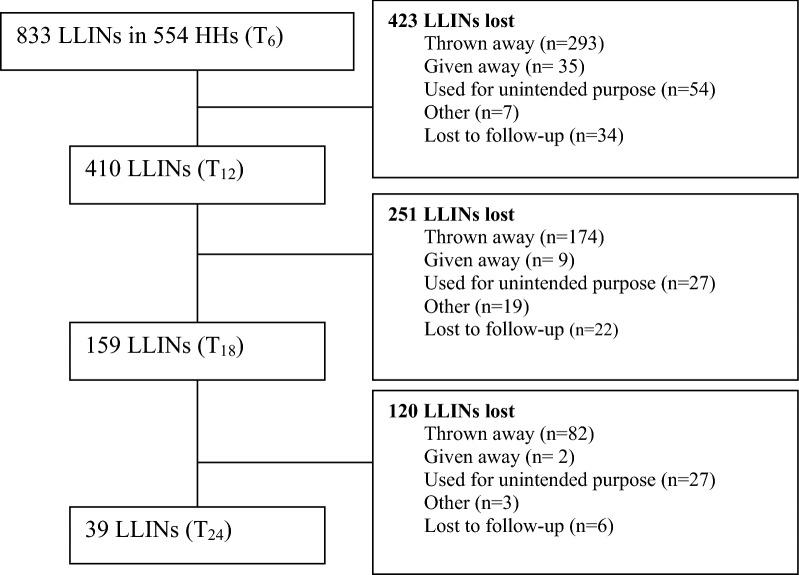
Flow diagram shows the number of LLINs enrolled at 6 months in south-central Ethiopia

The number of eligible LLINs, and those included for physical integrity evaluation at 6 months, as well as the number of LLINs found in the households during follow up are summarized in Fig. 3. The proportion of LLINs with a hole corresponding to the size categories 1–4 was 35.8% (298 of 833) after 6 months. This proportion increased to 79.5% (31 of 39) after 24 months of follow up. When the locations of holes were considered, the mean number of holes of any size was found to be higher in the lower half of the LLIN compared with the upper half or the roof. The median pHI increased from month 6 to 18, whereas the pHI decreased slightly at 24 months (Table 3).

**Table 3:** Proportion of long-lasting insecticide nets with holes, mean number of holes by location, and proportional hole index over time in Ethiopia

Characteristic	6 months N = 833	12 months N = 410	18 months N = 159	24 months N = 39
Holes (size categories 1–4), n (%)	298 (35.8)	204 (49.8)	107 (67.3)	31 (79.5)
Mean (SD) number of holes				
Lower half segment of nets	3.7 (12.7)	4.2 (12.0)	5.7 (13.3)	4.4 (6.6)
Upper half segment of nets	2.1 (8.1)	2.2 (4.6)	3.7 (8.1)	5 (9.2)
Roof segment of nets	1.2 (4.9)	1.8 (4.6)	2.6 (4.5)	3.3 (3.8)
GM pHI (95% CI)^a^	216 (167–279)	251 (195–323)	316 (234–427)	211 (117–378)
Median pHI (IQR)^a^	270 (48–993)	275 (88–843)	422 (173–775)	296 (77–604)

N: The total number of LLINs available for evaluation at each data collection period

*CI* Confidence interval, *IQR* interquartile range, *LLIN* long-lasting insecticidal net, *SD* standard deviation, *GM* geometric mean, *pHI* proportional hole index

^a^ Number of LLINs evaluated (n = 274 at 6 months, n = 170 at 12 months, n = 85 at 18 months, and n = 28 at 24 months)

The proportions of LLINs in the “good” and “acceptable” categories decreased with age, whereas LLINs in the “torn” category increased with age. The proportion of torn LLINs increased from 14.8% (123 of 833) to 23.1% (9 of 39) between 6 and 24 months. Only 39 LLINs were identified during follow up visits. Among these, only one LLIN was torn at 6 months, and the number of torn LLINs increased to nine (23.1%) after 24 months (Table 4).

**Table 4:** Proportion of long-lasting insecticide nets in good, acceptable, or torn condition over time, as defined by the proportional hole index in Ethiopia

Category defined by pHI	6 months N = 833 n (%)	12 months N = 410 n (%)	18 months N = 159 n (%)	24 months N = 39 n (%)
Good (0–64)	610 (73.2)	243 (59.3)	63 (39.6)	14 (35.9)
Acceptable (65–642)	100 (12.0)	82 (9.8)	45 (28.3)	16 (41.0)
Torn (> 643)	123 (14.8)	85 (20.7)	51 (32.1)	9 (23.1)
LLINs present at all follow-up visits, n = 39				
Good (0–64)	34 (87.2)	29 (74.4)	19 (48.7)	14 (35.9)
Acceptable (65–642)	4 (10.3)	6 (15.4)	13 (33.3)	16 (41.0)
Torn (> 643)	1 (2.6)	4 (10.3)	7 (17.9)	9 (23.1)

N: The total number of LLINs available for evaluation at each data collection period

*LLIN* long-lasting insecticidal net, *pHI* proportional hole index

### Predictors of physical integrity of LLINs

A bivariate proportional Cox regression analysis showed that using the LLIN during the night before the day of the survey, having a clean LLIN, and the presence of a cat in the house were all associated with the good physical integrity of LLINs. The presence of rats in the house and a household location of more than 1 km from a mosquito breeding site were associated with poor physical integrity. Multivariate analysis indicated that using a LLIN during the previous night (adjusted hazard ratio [HR] = 0.7; 95% CI 0.50–0.98), having a clean LLIN (adjusted HR = 0.4; 95% CI 0.30–0.60), and being in a household more than 1 km away from a mosquito breeding site (adjusted HR 1.8; 95% CI 1.2–2.6) were independent predictors of the physical integrity of LLINs (Table 5).

**Table 5:** Predictors of physical integrity of long-lasting insecticide nets over a 2-year follow-up period in Ethiopia

Variables	Net months observation	Number of torn LLINs	IR/100 NMO (95% CI)	Crude HR (95% CI)	Adjusted HR (95% CI)
Gender of head of household					
Male	5250	138	2.6 (2.2–3.1)	1.00	1.00
Female	2958	60	2.0 (1.5–2.5)	0.8 (0.6–1.0)	0.8 (0.6–1.3)
Educational status of head of household (n = 816)					
Illiterate	4458	108	2.4 (2.0–2.9)	1.0	NA
Read and write	822	19	2.3 (1.4–3.5)	1.0 (0.6–1.6)	
Primary	2010	44	2.2 (1.5–2.8)	0.9 (0.6–1.3)	
Secondary and above	786	18	2.3 (1.2–3.3)	1.0 (0.6–1.6)	
Wealth status					
Poor	2826	77	2.7 (2.1–3.3)	1.0	1.0
Middle	2640	62	2.3 (1.8–2.9)	0.9 (0.6–1.2)	1.0 (0.7–1.5)
Rich	2742	59	2.2 (1.6–2.7)	0.8 (0.6–1.1)	0.9 (0.6–1.4)
Type of bed					
Wooden bedframe	3786	86	2.3 (1.8–2.8)	1.0	NA
Stick or iron bedframe	924	23	2.5 (1.5–3.5)	1.1 (0.7–1.8)	
Mattress (with no bed frame)	2340	56	2.4 (1.8–3.0)	1.1 (0.8–1.5)	
Mat	1158	33	2.8 (1.9–3.8)	1.2 (0.8–1.9)	
LLIN hung up					
No	2358	64	2.7 (2.0–3.4)	1.0	NA
Yes	5850	134	2.3 (1.9–2.7)	0.8 (0.6–1.1)	
House has open eave gap (n = 820)					
No	6888	160	2.3 (2.0–2.7)	1.0	1.0
Yes	1158	38	3.3 (2.2–4.3)	1.4 (0.98–2.0)	1.0 (0.6–1.4)
LLIN used last night					
No	2496	75	3.0 (2.3–3.7)	1.0	1.0
Yes	5712	123	2.2 (1.8–2.5)	0.7 (0.5–0.96)*	0.7 (0.5–0.96)*
LLIN ever washed					
No	4992	109	2.2 (1.8–2.6)	1.0	1.0
Yes	3216	89	2.8 (2.2–3.3)	1.3 (1.0–1.7)	1.2 (0.9–1.7)
LLIN was clean					
No	4440	156	3.5 (3.0–4.1)	1.0	1.0
Yes	3768	42	1.1 (0.8–1.5)	0.3 (0.2–0.4)*	0.4 (0.3–0.6)*
Rats present in the house (n = 658)					
No	3900	73	1.9 (1.4–2.3)	1.0	1.0
Yes	3186	82	2.6 (2.0–3.1)	1.4 (1.02–1.92)*	1.1 (0.8–1.5)
Cat present in the house (n = 658)					
No	3648	94	2.6 (2.1–3.1)	1.0	1.0
Yes	3438	61	1.8 (1.3–2.2)	0.7 (0.5–0.9)*	0.8 (0.5–1.1)
Distance from mosquito breeding site (n = 833) (km)					
≤ 1	2844	51	1.8 (1.3–2.3)	1.0	1.0
> 1	5364	147	2.7 (2.3–3.1)	1.5 (1.1–2.1)*	1.8 (1.2–2.6)*
Family size (n = 833)					
≤ 5	3594	90	2.5 (2.0–3.0)	1.0	NA
> 5	4614	108	2.3 (1.9–2.8)	0.9 (0.7–1.2)	

*NMO* net months of observation, *IR* incidence rate, *LLIN* long-lasting insecticidal net, *HR* hazard ratio, *NA* not applicable when P < 0.25, *statistically significant at P < 0.05

### Functional survival

**Fig. 4:**
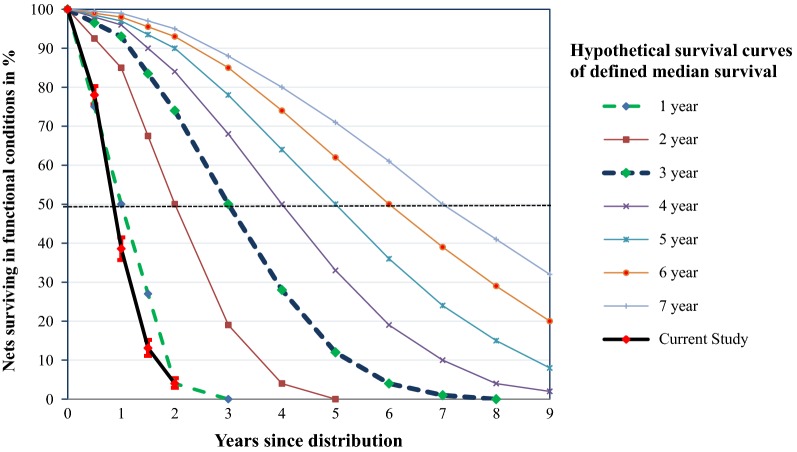
Functional survival of LLINs in south-central Ethiopia. The error bars indicate the 95% confidence interval

Observed functional survivals at different time points, compared with the reference NetCALC loss prediction curves, are shown in Fig. 4. Six months after distribution, 78.6% (95% CI 76.2–80.9) of the LLINs were functionally surviving. However, by month 24, only 4% (95% CI 2.9–5.4) had survived. The median (95% CI) survival time (time since distribution, in which 50% of LLINs were in a serviceable condition) was 12 (11.6–12.4) months. The observed functional survival was less than the 3-year serviceable model, being closer to the 1-year serviceable model (Fig. 4).

### Predictors of functional survivorship of LLINs

A multivariate proportional Cox regression model showed that having a clean LLIN (adjusted HR = 0.8; 95% CI 0.6–0.9) was an independent predictor of longer functional survival, whereas the distance of a household 1 km from a mosquito breeding site (adjusted HR = 1.3; 95% CI 1.1–1.6) was associated with the shorter functional survival of LLINs (Table 6).

**Table 6:** Predictors of functional survival of long-lasting insecticidal nets over a 2-year follow-up period in Ethiopia

Variable	Net month observation	Number of lost LLINs	IR/100 NMO (95% CI)	Crude HR (95% CI)	Adjusted HR (95% CI)
Gender of head of household (n = 1193)					
Male	9930	660	6.6 (6.1–7.2)	1.0	NA
Female	5262	333	6.3 (5.6–7.0)	1.0 (0.8–1.1)	
Educational status of head of household (n = 1170)					
Illiterate	8472	567	6.7 (6.1–7.2)	1.0	1.0
Read and write	1554	99	6.4 (5.1–7.6)	0.9 (0.7–1.1)	0.9 (0.7–1.3)
Primary	3606	227	6.3 (5.5–7.1)	0.9 (0.8–1.1)	1.0 (0.9–1.3)
Secondary and above	1308	79	6.0 (4.7–7.4)	0.9 (0.7–1.1)	0.9 (0.7–1.2)
Wealth status (n = 1184)					
Poor	5244	363	6.9 (6.2–7.6)	1.0	1.0
Middle	4872	312	6.4 (5.7–7.1)	0.9 (0.8–1.0)	1.0 (0.8–1.2)
Rich	4968	312	6.3 (5.6–7.0)	0.9 (0.8–1.0)	0.9 (0.8–1.2)
Open eave (n = 1176)					
No	12,480	819	6. 6 (6.1–7.0)	1.0	NA
Yes	2460	166	6.7 (5.7–7.8)	1.0 (0.9–1.2)	
LLIN used last night (n = 833)					
No	3468	237	6.8 (6.0–7.7)	1.0	1.0
Yes	7746	459	5.9 (5.4–6.5)	0.8 (0.7–0.97)*	1.0 (0.7–1.0)
LLIN ever washed (n = 833)					
No	6876	423	6.2 (5.6–6.7)	1.0	
Yes	4338	273	6.3 (5.5–7.0)	1.0 (0.9–1.2)	NA
LLIN was clean (n = 833)					
No	6012	418	7.0 (6.3–7.6)	1.0	1.0
Yes	5202	278	5.3 (4.7–6.0)	0.7 (0.6–0.8)*	0.8 (0.6–0.9) *
Rats present in the house (n = 858)					
No	6462	382	5.9 (5.3–6.5)	1.0	1.0
Yes	5316	334	6.3 (5.6–7.0)	1.1 (1.0–1.3)	1.1 (0.9–1.3)
Cat present in the house (n = 858)					
No	6102	378	6.2 (5.6–6.8)	1.0	
Yes	5676	338	6.0 (5.3–6.6)	1.0 (0.8–1.1)	NA
Distance from mosquito breeding site (n = 1193) (km)					
≤ 1	5058	311	6.1 (5.5–6.8)	1.0	1.0
> 1	10,134	682	6.7 (6.2–7.2)	1.2 (1.1–1.3)*	1.3 (1.1–1.6)*
Household population size (n = 1193)					
≤ 5	6264	392	6.3 (5.6–6.9)	1.0	1.0
> 5	8928	601	6.7 (6.2–7.3)	1.1 (1.0–1.3)	1.1 (0.9–1.3)

*NMO* net month observation, *IR* incidence rate, *LLIN* long-lasting insecticidal net, *HR* hazard ratio, *NA* not applicable when P < 0.25, *statistically significant at P < 0.05

### Bio-efficacy

A total of 120 LLINs were tested using WHO cone bioassays over a 2-year period. The GM of 60-min KD rates was greater than 90% in all four surveys. The GM of 24-h mortality rates was below 80% in the second year with 76.6% (95% CI 71.0–82.6) at 12 months and 69.4% (95% CI 59.4–80.9) at 24 months (Table 7). Statistically significant differences were observed both in 60-min KD rates, and 24-h mortality rates between 12 and 18 months and between 12 and 24 months (Table 8).

**Table 7:** Geometric means of 1-h knockdown and 24-h mortality of mosquitoes in Ethiopia

Survey (months)	Number of LLINs	60-min KD GM (95% CI)	24-h mortality GM (95% CI)
6	30	94.1 (87.1–100)	81.1 (67.7–97.0)
12	30	99.9 (99.7–100)	89.5 (87.2–91.8)
18	30	93.9 (90.0–98.1)	76.6 (71.0–82.6)
24	30	94.1 (91.3–97.1)	69.4 (59.4–80.9)

*CI* confidence interval, *GM* geometric mean, *KD* knockdown, *LLIN* long-lasting insecticidal net

**Table 8:** Mean differences in the proportions of knockdowns and mortality of mosquitoes in Ethiopia

Variable	Between months	Mean difference (95% CI)^a^	P value
Knockdown	12 and 18	5.4 (0.4–10.4)	0.029
	12 and 24	5.5 (1.7–9.3)	0.002
Mortality	12 and 18	11.7 (4.1–19.2)	0.001
	12 and 24	15.4 (3.1–27.8)	0.008

*CI* confidence interval

^a^ One-way ANOVA assuming unequal variance used

The proportion of LLINs meeting the WHO pesticide evaluation scheme criteria at different time points is presented in Table 9. At 6 months, the proportion of LLINs meeting the criteria of effective bio-efficacy was 90% (95% CI 72.5–96.8), and this proportion decreased to 80% (95% CI 61.5–90.9) at 24 months. However, LLINs met the criteria of effective bio-efficacy in all study periods.

**Table 9:** Proportion of long-lasting insecticidal nets meeting WHO pesticide evaluation scheme criteria effective (1-h knockdown ≥ 95% or 24-h mortality ≥ 80%) in Ethiopia

Age of LLINs (months)	Number evaluated	n (%), 95% CI
6	30	27 (90.0), 72.5–96.8
12	30	30 (100)
18	30	25 (83.3), 65.1–93.1
24	30	24 (80.0), 61.5–90.9

*CI* confidence interval, *LLINs* long-lasting insecticidal nets, *WHO* World Health Organization

## Discussion

Low functional survivorship of LLINs was observed in south-central Ethiopia. The data show that most LLINs survive for approximately 1 year. High attrition rates due to discarding and the poor physical integrity of LLINs were the major causes of low functional survivorship. The LLINs were found to be effective against malaria-transmitting mosquitoes and met the criteria of optimal effectiveness of bio-efficacy up to month 24.

Previous studies have used cross-sectional study designs to evaluate the durability of LLINs in Ethiopia [17, 21]. Because of the design, these studies could not quantify attrition, functional survivorship, and changes in the physical integrity of LLINs over time. The current study has addressed these limitations. Unlike previous follow-up studies [8, 13, 39], this study followed all LLINs in selected households to avoid selection bias and potential observer effects (Hawthorne effect) in which users might treat the net under observation differently than nets not under observation.

This study had some limitations. The prospective nature of the study may have influenced the user to keep their LLINs longer because they were being observed. However, because the attrition rate was much higher than expected, this potential limitation is less likely to have influenced the results. The functional survival time of LLINs may have been overestimated because LLINs could be lost at any time during the 6-month follow-up period. There may have also been recall bias, as people may not have correctly remembered what had happened to their LLINs over the previous 6 months. Furthermore, it was difficult to trace the reason for LLIN loss when more than one LLIN was lost within the same household.

As expected, the physical integrity of LLINs deteriorated over time. The proportion of LLINs with a hole size 0.5 cm and larger (36–80% of LLINs) between 6–24 months was comparable to other studies in Ethiopia (54.5–85.5%) between 6–20 months [17], and in Zambia (60.2–87.2%) between 12–24 months [8]. The observed high number of holes in the lower half of the nets was also consistent with the findings of previous studies [8, 10, 17, 40]. The previous study reported that using nets over a reed mat was significantly associated with larger holes in the lower half of the nets [8].

Using the LLIN the night before the survey was associated with the good physical integrity of LLINs. Net use and having good physical integrity might have a bi-directional association. The users might keep their in-use nets from physical damage. Conversely, the users might prefer to use intact nets more than damaged nets. Having a clean LLIN was another predictor of the good physical integrity of LLINs in this study. This finding also might be due to the tendency of users to keep an intact net clean for prolonged use compared with damaged nets. Moreover, the presence of kitchens inside the house or using firewood as a cooking fuel could make the nets dirty [13]. Thus, dirty nets may be frequently washed, and could lose their physical integrity. Proximity to a mosquito breeding site was a significant predictor of physical integrity. The LLINs in households that lived more than 1 km away from potential vector breeding sites were more likely to be damaged than households located within a 1 km radius. This finding could be explained by nets being less valued in areas with a lower perceived risk of mosquito bites and malaria infection. Evidence from the qualitative study showed the tendency of owners far from potential mosquito breeding sites to misuse nets [14].

Six months after distribution, the functional survivorship of LLINs was 78.6%. This percentage is lower than that reported in a study in Benin (93%) [13]. One potential explanation for this difference is that all LLINs presented in the household, including torn nets, were considered to have survived in the Benin study. Moreover, the percentage of surviving nets in this study is lower than the NetCALC model 3-year serviceable prediction value of 96.5% [41]. After 12 months, net survivorship further decreased to 38.6%, lower than the 72% reported from Benin [13] and 90.4% in Zambia [8]. In our area, the greatest loss (40%) occurred during the 6- to 12-month period after LLIN distribution, and is probably related to the unusually dry and warmer weather that followed the El Nino in 2015 [30]. Moreover, the marked decline in the incidence of malaria in the study area (only 37% of predistribution incidence) [42] could have indirectly affected the survivorship of LLINs by decreasing the perceived risk of malaria infection. After 24 months, the functional survival was only 4%, which is substantially lower than the expected 75% by the NetCALC 3-year serviceable prediction model [41]. In general, the functional survival of LLINs in the current study is comparable to a 1-year serviceable prediction model, in which 4% of LLINs are predicted to survive after 2 years [36, 41].

In addition to the unexpected weather conditions and a decline in the incidence of malaria, the behaviour of the net users could play a role in high attrition rate and low functional survivorship. A qualitative study done on the same households as our study showed that many informants believed that the LLINs would not serve more than 1 year. The users claimed the LLINs could lose their insecticidal effect after 6 months by mentioning that the nets “stopped killing bugs.” Washing LLINs several times was also believed to cause a loss of insecticides [14]. As explored by this qualitative study, after 1 year most of the LLINs were misused. However, this finding was not supported by the current study, as 64.2% of reported net loss was due to disposal. There could be a possible social desirability bias, because people did not report the misuse of LLINs in the current study. There is also a possibility that the LLINs were used for agricultural purposes, such as grain storage and transportation from the field, as well as the separation of grains from their chaffs, before being discarded as explored by the qualitative study [14]. Moreover, a low level of knowledge and a low positive perception towards net care and repair in Ethiopia may have also played a role in the observed high attrition, poor physical integrity and lower functional survival of LLINs [15].

In this study, having a clean LLIN was found to be associated with a longer functional survival time. This could be due to the behaviour of the owners, who would like to use LLINs for a prolonged time and thus keep the nets clean. A result from a qualitative study showed that nets become dirty from excessive smoke from indoor cooking stoves or fires, which leads the users to discard the nets prematurely or misuse them [14]. The LLINs in households living more than 1 km from potential vector breeding sites were less likely to survive. This could be related to a higher perceived risk of mosquito bites and malaria infection among net owners living closer to a vector breeding site [43, 44]. In this study, neither using the net the night before the survey nor having ever washed the net was associated with functional survival of LLINs. However, a previous study observed an association between using the net the night before the survey and a longer survival time, and an association between having ever washed the net and a shorter survival time [8].

Previous studies have reported that the bio-efficacy of the LLIN is correlated with the concentration of the insecticide [8, 21]. In the current study, PermaNet^®^ 2.0 LLINs met the WHO pesticide evaluation scheme criteria of bio-efficacy (at least 80% of the sampled LLINs effective in a WHO cone test) after 24 months [34], which was in agreement with other similar studies [21, 39] and higher than a result reported by Tan et al. [8].

In general, our results suggested that the survivorship of LLINs after 2 years was low compared with the prediction of the NetCALC model (4% vs 75%). This finding raises a serious concern about the programmatic assumption of the 3-year LLIN replacement cycle. Therefore, we suggest that nationally representative LLIN durability studies should be conducted to estimate the correct LLIN replacement cycle. Meanwhile, national malaria control programs should closely work with manufacturers to develop stronger and more durable LLIN products. Moreover, strengthening the behaviour change communication messages on net care and repair, as well as the proper use of LLINs, may help to improve the durability of LLINs.

## Conclusions

The study results suggested that the serviceable time of LLINs is 1 year, as a “3-year” serviceable assumption was unrealistic in this study community. Consequently, stronger and more efficient LLINs need to be developed for conditions similar to those studied here. After all, many parts of Ethiopia exhibit conditions similar to those at this study site. Because this study was conducted on one brand of LLIN and in one area only, the findings may not be extrapolated to other brands and people living in different topographic and socioeconomic settings. Therefore, more research still needs to be conducted to generalize the findings to the country level.

## Abbreviations

AHR: adjusted hazard ratio
CI: confidence interval
GM: geometric mean
KD: knockdown
IQR: interquartile range
IRS: indoor residual spray
LLIN: long-lasting insecticidal net
MD: mean difference
PCA: principal component analysis
pHI: proportional hole index
WHO: World Health Organization
