# Correction to: Bed nets used to protect against malaria do not last long in a semi-arid area of Ethiopia: a cohort study

**DOI:** 10.1186/s12936-019-2772-4

**Published:** 2019-04-19

**Authors:** Tarekegn Solomon, Eskindir Loha, Wakgari Deressa, Meshesha Balkew, Taye Gari, Hans J. Overgaard, Bernt Lindtjørn

**Affiliations:** 1 School of Public and Environmental Health, Hawassa University, Hawassa, Ethiopia; 2 Department of Preventive Medicine, School of Public Health, College of Health Sciences, Addis Ababa University, Addis Ababa, Ethiopia; 3 Aklilu Lemma Institute of Pathobiology, Addis Ababa University, Addis Ababa, Ethiopia; 4 Norwegian University of Life Sciences, Ås, Norway; 5 Centre for International Health, University of Bergen, Bergen, Norway

## Correction to: Malar J (2018) 17:239 10.1186/s12936-018-2391-5

Following publication of the original article [1], the author has flagged errors that affect the scientific content of the article.

The errors concern Table 2 (page 6), Table 5 (page 9) and Table 6 (page 11) of the published article.

Firstly, with concern to Table 2, the errors concern the ‘Totals’ provided in the table. Please find below (the correct version of) the table, with the corrected ‘Totals’ data highlighted in italics:

**Table 2:** Reasons for loss of long-lasting insecticide nets over a 2-year follow-up period in Ethiopia

Reason for LLIN loss	6 months	12 months	18 months	24 months	Total
	n (%)	n (%)	n (%)	n (%)	n (%)
Known outcome of attrition or torn					
Thrown away	102 (40.0)	301 (71.8)	173 (74.9)	62 (70.5)	638 (64.2)
Used for something else	30 (11.8)	58 (13.8)	28 (12.1)	22 (25.0)	138 (13.9)
Torn (pHI > 643)	123 (48.2)	60 (14.3)	30 (13.0)	4 (4.5)	217 (21.9)
Total	*255* (100)	*419* (100)	*231* (100)	*88* (100)	993 (100)
Unknown outcome of attrition					
Given away	260 (76.7)	44 (45.8)	11 (20.0)	1 (12.5)	316 (63.5)
Lost to follow-up^a^	52 (15.3)	43 (44.8)	26 (47.2)	5 (62.5)	126 (25.3)
Stolen	8 (2.4)	9 (9.4)	3 (5.5)	2 (25.0)	22 (4.4)
Unknown reasons	6 (1.8)	0 (0.0)	1 (1.8)	0 (0.0)	7 (1.4)
Other^b^	13 (3.8)	0 (0.0)	14 (25.5)	0 (0.0)	27 (5.4)
Total	*339* (100)	*96* (100)	*55* (100)	*8* (100)	498 (100)
*Overall total*	*594*	*515*	*286*	*96*	*1491*

*LLIN* long-lasting insecticidal net

^a^Family moved to other location, family not at home, refusal to participate

^b^Sold or destroyed by fire

Regarding **Tables** **5 and 6**, the error on the footnotes of the published tables is: “**NA not applicable when P < 0.25”.**

The footnotes on **Tables** **5 and 6** should read as: “**NA not applicable when P > 0.25”.**
